# Information-driven attentional capture

**DOI:** 10.3758/s13414-024-03008-z

**Published:** 2025-02-19

**Authors:** Alenka Doyle, Kamilla Volkova, Nicholas Crotty, Nicole Massa, Michael A. Grubb

**Affiliations:** 1 Trinity College, Hartford, CT USA; 2 Mass General Brigham, Boston, MA USA

**Keywords:** Attention, Attentional capture, Attention in learning

## Abstract

Visual attention, the selective prioritization of sensory information, is crucial in dynamic, information-rich environments. That both internal goals and external salience modulate the allocation of attention is well established. However, recent empirical work has found instances of experience-driven attention, wherein task-irrelevant, physically non-salient stimuli reflexively capture attention in ways that are contingent on an observer’s unique history. The prototypical example of experience-driven attention relies on a history of reward associations, with evidence attributing the phenomenon to reward-prediction errors. However, a mechanistic account, differing from the reward-prediction error hypothesis, is needed to explain how, in the absence of monetary reward, a history of target-seeking leads to attentional capture. Here we propose that what drives attentional capture in such cases is not target-seeking, but an association with instrumental information. To test this hypothesis, we used pre-cues to render the information provided by a search target either instrumental or redundant. We found that task-irrelevant, physically non-salient distractors associated with instrumental information were more likely to draw eye movements (a sensitive metric of information sampling) than were distractors associated with redundant information. Furthermore, saccading to an instrumental-information-associated distractor led to a greater behavioral cost: response times were slowed more severely. Crucially, the distractors had equivalent histories as sought targets, so any attentional differences between them must be due to different information histories resulting from our experimental manipulation. These findings provide strong evidence for the information history hypothesis and offer a method for exploring the neural signature of information-driven attentional capture.

**Supplementary information:**

The online version contains supplementary material available at 10.3758/s13414-024-03008-z.

## Introduction

In dynamic, information-rich environments, the selective prioritization of sensory information is crucial. A foundational question in the study of visual attention has been: *What determines the target of prioritization?* It has been firmly established that both the internal goals of the observer and the physical salience of an external stimulus modulate the allocation of attention, with this dichotomy serving the field well for many decades (Carrasco, 2011).

But this theoretical framework is incomplete. Extensive empirical work has shown that task-irrelevant, physically non-salient stimuli modulate the allocation of attention in ways that are contingent on an observer’s unique history (Awh et al., 2012), with multiple, distinct components theorized to constitute instances of “experience-driven attention” (Anderson et al., 2021). The prototypical example of experience-driven attention, value-driven attentional capture (VDAC; Anderson et al., 2011), relies on a history of reward associations. VDAC is engendered by pairing an arbitrary stimulus feature (e.g., color) with the receipt of monetary reward during a “training phase.” In a subsequent “test phase,” non-salient visual distractors rendered in colors that predicted reward during the training phase capture attention by slowing response times (RTs) and drawing eye movements. Attention is modulated in a reward-magnitude-dependent manner: distractors associated with a higher-value reward capture more attention than those associated with a lower-value reward (Anderson & Halpern, 2017). Neuroimaging data have implicated dopaminergic neurons in the generation of VDAC (Anderson et al., 2016), with a preponderance of evidence suggesting that the phenomenon is attributable to reward-predictions and errors thereof (Anderson et al., 2021).

Recent work from our lab used behavioral methods to further test the reward-prediction account of VDAC by manipulating the temporal presentation of reward-predicting stimuli (Massa et al., 2024). In a traditional VDAC training phase, the color of the search target indicates upcoming reward magnitude; therefore, search targets are theorized to elicit magnitude-dependent reward predictions prior to reward acquisition. In Massa et al. (2024), we modified the training phase to include a “pre-cue” which preceded the visual search array and provided target-color information with varying amounts of certainty. One pre-cue provided precise target-color information, becoming the earliest reliable predictor of upcoming reward magnitude (the “reliable” pre-cue). Previous work on dopamine-mediated higher-order conditioning (Schultz, 2015) suggests that the reward prediction should be transferred to the pre-cue, as it was the earliest reward-predicting stimulus. Such a shift should also abolish the target’s role as a reward predictor. Opposing the reliable pre-cue was the “unreliable” pre-cue, which provided imprecise information and allowed unreliably pre-cued targets to remain the earliest reward-predicting stimuli and retain their reward associations.

We predicted that, in the test phase, attention would be modulated by an interaction between cue type and reward magnitude. Specifically, we hypothesized that the difference in attentional capture between a high-reward predicting, unreliably pre-cued distractor and a low-reward predicting, unreliably pre-cued distractor (i.e., VDAC value-dependency) would exceed that observed between high-reward and low-reward predicting distractors that had been reliably pre-cued. Our results partially aligned with these predictions (Massa et al., 2024) in that unreliably pre-cued distractors slowed test phase RTs and drew more eye movements relative to reliably pre-cued distractors (i.e., a main effect of information reliability on attentional capture) without an accompanying interaction (however, for evidence that such an interaction is possible using a similar protocol, see Mahlberg et al., 2024,). Furthermore, in Massa et al. (2024), there was no main effect of reward magnitude. In sum, those results suggested that our experimental manipulation increased the saliency of the information content of target color, and in doing so, caused information to supersede reward.

In Massa et al. (2024), both reliably and unreliably pre-cued distractors had equivalent histories as sought targets (Anderson et al., 2021), but by providing target-color information in advance, we successfully manipulated the degree to which they elicited the reflexive allocation of experience-driven attention. Such attentional differences between the two conditions might be directly attributable to their different information histories. This is an exciting prospect, as it could provide a mechanistic account, separate from reward-predictions, to explain the reflexive allocation of experience-driven attention currently attributed to “history as a sought target” (Anderson et al., 2021) in designs that removed monetary reward altogether (e.g., Grubb & Li, 2018; Kim & Anderson, 2019; Sha & Jiang, 2016). In the present study, we utilize the same information manipulation from Massa et al. (2024) but remove monetary reward entirely to ensure that any findings are due only to experience-driven attention and not confounded by shifts of value-driven attention.

**Fig. 1:**
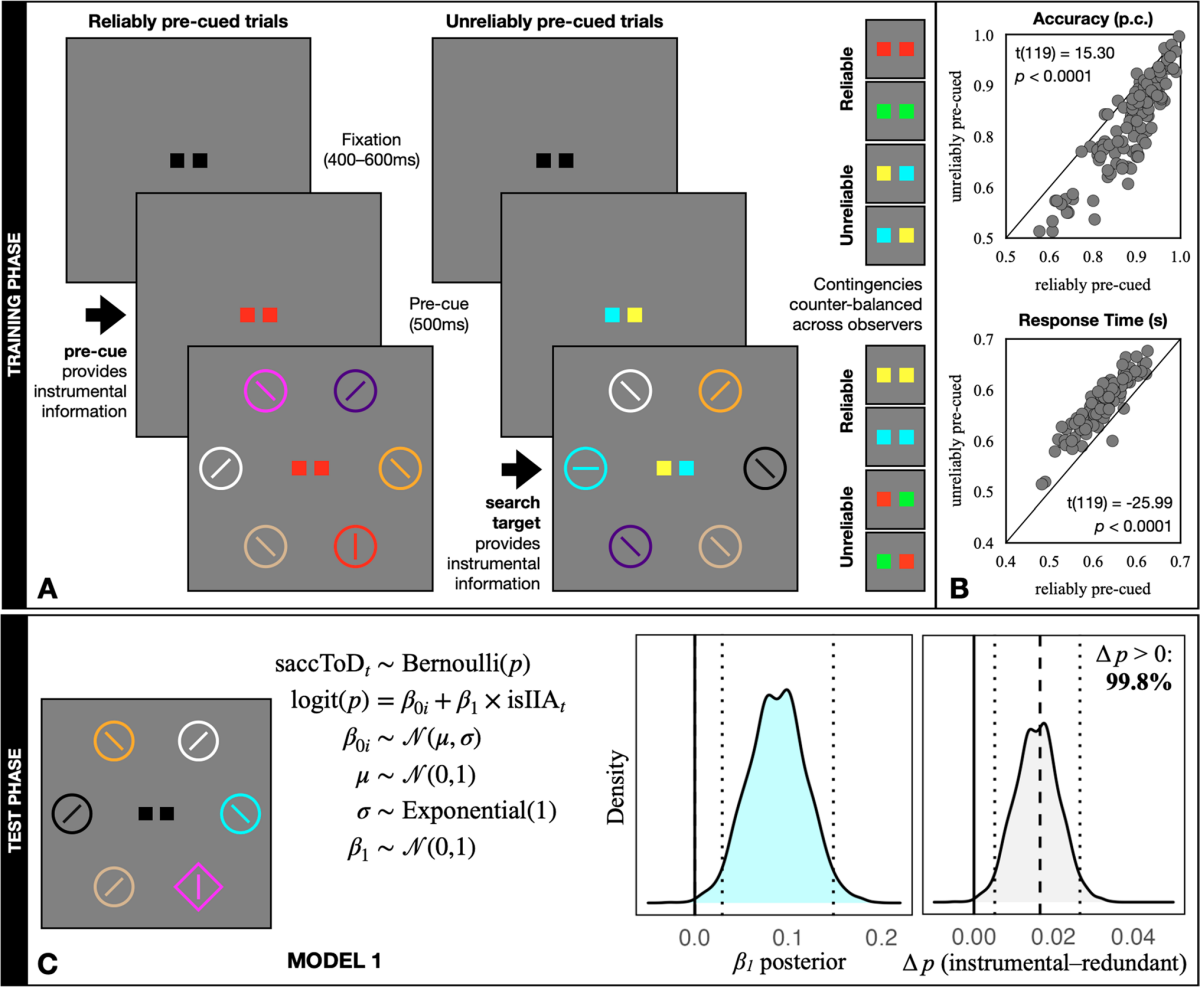
**A **Training phase task and information manipulation. Observers have 800 ms to search for a color-defined target and report the orientation of the line inside (horizontal or vertical). Half the trials are pre-cued with reliable information about target color (target matches pre-cue color) and half are pre-cued with unreliable information (target matches one of two pre-cue colors). **B **Training phase results. Proportion correct (**top**) and mean response time for correct trials (**bottom**) for each pre-cued condition; each dot is an individual participant; the outcome of a paired *t*-test is reported in each scatterplot. **C **Test phase task and results. Observers have 1,200 ms to search for the shape singleton and report the orientation of the line inside (horizontal or vertical). Half the trials contain an information-associated distractor (instrumental-information-associated or redundant-information-associated). Dotted lines on posterior distributions indicate 95% credible intervals, the dashed line indicates the change in proportion of saccades calculated directly from the data. See main text for additional details

Here we propose and test the hypothesis that what drives attentional capture in the absence of monetary reward is not a history of target-seeking, but *information history*. A growing literature on the neuroscience of information-seeking suggests that information can be a strong modulator of behavior and neurophysiology (Charpentier & Cogliati Dezza, 2022; Murayama, 2022). If the color of a search target is always one of two colors (e.g., red or green), but the exact color is unknown at the start of the trial, then the appearance of the search target provides trial-to-trial information. In such studies, information is instrumental, as it allows observers to localize the target and make the required orientation judgment. Thus, target-defining colors become associated with a history of providing instrumental information. In order to manipulate the information history of each target-defining color in the current study, we implemented the pre-cues used in Massa et al. (2024). We reasoned that if a reliable pre-cue provided precise target-color information in advance of the search array for one pair of target colors (Fig. 1A, reliably pre-cued trials), it would render the target-color information provided by that target redundant. Inversely, if an unreliable pre-cue provided imprecise information for a different pair of target colors (Fig. 1A, unreliably pre-cued trials), it would render the information provided by that target instrumental. Crucially, history as a sought target was held constant, and we removed monetary reward entirely. We then tested for experience-driven attentional capture in a typical test phase, wherein half of the trials contained an information-associated distractor. Of these trials, half of them contained a distractor associated with *instrumental* information (i.e., rendered in colors used for *unreliably* pre-cued targets in the training phase). The other half of the distractor-present trials contained a distractor associated with *redundant* information (i.e., rendered in colors used for *reliably* pre-cued targets during training).

In Massa et al. (2024), oculomotor data provided the most sensitive metric of attentional capture. Given the similarity of the current design with that used in Massa et al. (2024) and the observation that eye movements directly reflect information sampling (Gottlieb et al., 2013), here we operationalized attentional prioritization as a saccade to the location of either information-associated distractor. We then compared saccades made to distractors associated with redundant information to saccades made to distractors associated with instrumental information. Given that the two types of information-associated distractors had equivalent histories as sought targets, a difference between them can only be attributed to their different information histories.

## Methods

### Overview

Participants completed a modified version of the experimental protocol carried out in Massa et al. (2024), with monetary reward replaced by trial-to-trial task accuracy-based feedback. The experiment consisted of 960 total trials in an approximately 1.5-h session.

### Observers

One hundred and thirty-one observers participated in the study; all observers self-reported typical color vision. Two observers participated in Massa et al. (2024) and were inadvertently allowed to participate in the current study; both were excluded to ensure that no lingering associations impacted performance. Data from nine additional participants were excluded from all reported analyses for performing at chance accuracy levels (a detailed exclusion process is available in the Online Supplemental Material (OSM)). These exclusions resulted in 120 participants for data analysis (age: mean = 19.47 years, range = 18–26 years; gender: 83 F, 35 M, two non-binary). This study received ethical approval from the Trinity College Institutional Review Board, and informed consent was obtained for each participant.

### Tasks and experimental manipulations

In the training phase, participants searched for a color-defined target (red, green, cyan, or yellow) within a visual search array and reported the orientation of the line contained within the target (horizontal or vertical). The search array was preceded by a pre-cue: a color change at fixation providing either one target-color option (reliable) or two target-color options (unreliable). They then received feedback about the accuracy of the orientation judgment (correct, incorrect, or too slow). There were 480 trials in the training phase, delivered as five blocks of 96 trials. This stage was followed by the test phase, in which participants searched for a shape-defined target (a diamond among circles or a circle among diamonds) and completed the same line-orientation discrimination task, followed by task accuracy feedback. On half of the trials, one of the five distractor elements was rendered in a color (red, green, cyan, or yellow) that was associated with information (instrumental or redundant) in the training phase. There were 480 trials in the test phase, delivered as five blocks of 96 trials.

### Eye tracking

Eye movements were recorded using an EyeLink 1000 infrared-video eye tracker (Eyelink, SR Research, Ottawa, Ontario, Canada). A 9-point calibration routine was performed before each experimental phase.

### Detailed methods

Given the space constraints of this article type, detailed methods are provided in the Online Supplemental Material (OSM).

## Results

In the training phase, participants were sensitive to the information content of the pre-cues. When pre-cues signaled reliable information, orientation discrimination judgments were more accurate and RTs were faster than when pre-cues signaled unreliable information (Fig. 1B, Table 1). This pattern of results is consistent with a vast literature on feature-based attention (Carrasco, 2011).

**Table 1:** Mean (standard deviation) for training-phase conditions

	Reliably pre-cued	Unreliably pre-cued
Response time (ms)	568.09 (38.36)	606.52 (37.81)
Accuracy (proportion correct)	0.8551 (0.09)	0.7798 (0.12)

In the test phase, distractors associated with a history of providing instrumental information (i.e., rendered in colors used for *unreliably* pre-cued targets in the training phase) were more likely to draw a saccade than were distractors associated with redundant information (i.e., rendered in colors used for *reliably* pre-cued targets during training). For each participant, we calculated the proportion of trials in which a saccade was made to (i) the location of an instrumental-information-associated distractor, (ii) the location of a redundant-information-associated distractor, and (iii), for the distractor-absent trials, the location of a “non-target” stimulus (we divided this number by 5 to account for the fact that there were five potential non-target stimuli that could be attended). A one-way ANOVA on these values, with distractor condition as a within-subject repeated-measures factor, indicated a significant main effect (*F*(2,236) = 108.7, *p* < 0.0001, see Table 2 for condition means). Follow-up paired t-tests demonstrated that both instrumental- and redundant-information-associated distractors increased the proportions of saccades to their locations, relative to the distractor-absent condition (both *t*s > 11.01, both *p*s < 0.0001). To assess our hypothesis that information modulates experience-driven attention, we compared the two conditions where “history as a sought target” was matched and information history was manipulated: instrumental- and redundant-information-associated distractors. In support of our hypothesis, the presence of an instrumental-information-associated distractor increased the proportion of saccades to its location, relative to a redundant-information-associated distractor (*t*(118) = 2.36, *p* = 0.0200, two-tailed).^1^

**Table 2:** Mean (standard deviation) for test-phase conditions

	Distractor-absent	Reliably pre-cued	Unreliably pre-cued
Proportion of saccades to distractor	0.1389 (0.02)	0.2336 (0.10)	0.2535 (0.10)
Accuracy (proportion correct)	0.8660 (0.09)	0.8610 (0.10)	0.8626 (0.10)
Response time (ms)	725.98 (76.45)	724.92 (74.73)	728.06 (74.47)

Eye movements proved to be the most sensitive metric of attentional capture. Neither accuracy (*F*(2,238) = 1.001, *p* = 0.369) nor mean RT (*F*(2,238) = 1.49, *p* = 0.227) differed by condition^2^ (Table 2). That said, individual differences in RT were correlated with individual differences in oculomotor capture: the more that instrumental-information-associated distractors drew saccades (relative to redundant-information-associated distractors) the more that instrumental-information-associated distractors slowed RTs (relative to redundant-information-associated distractors) (*r*(117) = 0.2572, *p* = 0.0047).

Readers are likely to be familiar with using an ANOVA and *t*-tests to statistically compare experimental conditions, and as reported in the previous paragraph, evidence for our primary hypothesis was obtained with this frequentist approach. That said, analyzing oculomotor data at the subject level cannot account for the number of discrete trials that underlie each unique proportion, which varies across participants and across conditions (see *Saccadic variability *in the OSM*Results* for more on this point). Furthermore, some readers may prefer analyses that employ Bayesian statistics to make inferences and draw conclusions. To simultaneously address both concerns, we fit a Bayesian model to the trial-level data. Specifically, we used generalized linear mixed-effects modeling with hierarchal Bayesian estimation to assess differences in the amount of overt attention allocated to the test phase distractors (instrumental-information-associated or redundant-information-associated, with distractor-absent trials excluded). For readers less familiar with this approach, see *Data analysis*, oculomotor capture, Bayesian modeling in the OSM*Methods*.

We modeled the probability that a saccade was made to the location of the distractor on a given trial (*saccToD*_*t*_, Model 1, Fig. 1C). In this model, β_1_ quantifies the degree to which the presence of an instrumental-information-associated distractor (*isIIA*) changes the probability that a saccade is made to its location (a fixed effect) relative to a given individual’s probability of a saccade to a redundant-information-associated distractor (a random effect, β_0i_). We used uninformative prior distributions so as not to bias the outcome in either direction (OSM*Methods*), and we estimated posterior distributions using Hamiltonian Monte Carlo (McElreath, 2020).

Consistent with the frequentist approach, distractors associated with instrumental information were more likely to draw a saccade than were distractors associated with redundant information. The mean of the posterior distribution of β_1_ was positive (0.0883), its 95% credible interval did not contain zero (0.0294–0.1484), and 99.8% of its density exceeded zero (Fig. 1C, cyan). To better visualize the effect, we used the Bayesian model results to calculate the probability of a saccade made to each distractor type (redundant-information: 0.2365, instrumental-information: 0.2523) and plotted the posterior distribution of this difference (Fig. 1C, gray).^3^ In sum, distractors associated with a history of providing instrumental information captured overt attention more strongly than did distractors with a history of providing redundant information, despite equivalent histories as sought targets during the training phase.

## Discussion

In this study, we isolated the impact of information history on the reflexive allocation of visual attention using an atypically large sample (120 participants). As in our previous work (Massa et al., 2024), we used reliable and unreliable pre-cues in the training phase to manipulate the instrumentality of target-color information. Unlike our approach in Massa et al. (2024), we replaced trial-to-trial monetary reward in the training phase with task accuracy-based feedback in order to avoid any influence of reward associations and focus exclusively on experience-driven attention. We tested the hypothesis that information history, rather than a history as a sought target per se, drives attentional capture in the absence of monetary reward. In the test phase, we found that distractors associated with a history of providing instrumental information were more likely to draw saccades than were distractors associated with a history of providing redundant information. Given that the two types of information-associated distractors had equivalent histories as sought targets,^4^ these differences must be due to their different information histories.

In totality, our results establish the importance of collecting oculomotor data. The only methodological difference between Massa et al. (2024) and the current study was the use of reward-based feedback in the former and accuracy-based feedback in the latter. That we found a straightforward impact of distractors on RT in Massa et al. (2024) but failed to find a direct effect on RT here suggests that the underlying mechanisms between value-driven and information-driven attentional capture are distinct. That said, we did observe an impact of information history on RT in the current study, but it manifested as an interaction with eye movements to the distractors themselves (*RT and oculomotor capture, Bayesian modeling* in OSM*Results*). Had we not collected eye-tracking data in the current study, we would have been unable to detect robust effects of information history on the allocation of overt visual attention.

Additional questions remain surrounding the ability of the redundant-information-associated distractor to capture some degree of overt attention, relative to the distractor-absent condition. Although these distractors were not associated with a history of providing instrumental information about target *color* when they appeared as targets in the training phase, they did provide instrumental information about target *location.* Future work will be needed to confirm whether such instrumental location information modulates subsequent attentional capture, but this possibility aligns with the information-driven attention hypothesis. Importantly, information about target location was matched (i.e., equally uncertain) between redundant- and instrumental-information-associated distractors in the current study.

Our behavioral results suggest a potential role for brain networks that have been implicated in information seeking. Future neuroimaging work can test the possibility that distractors associated with a history of instrumental information evoke activity in a “shared network” that encodes the value of both information and other rewards (e.g., money), or, alternatively, an “independent network” that has been implicated in uncertainty reduction (Charpentier & Cogliati Dezza, 2022; Murayama, 2022). Extant fMRI data suggest that both are plausible hypotheses. In a previous study, Kim and Anderson (2019) compared BOLD (blood oxygenation level dependent) activity in the “value-driven attentional network” in observers who received unrewarded feedback during training with those from Anderson et al. (2014), who received reward feedback during training, using a mask that was generated by an independent dataset (Anderson, 2017). Despite a hemispheric interaction, with more right hemisphere activation following unrewarded training, both datasets showed distractor-evoked activity in the value-driven attentional network. Kim and Anderson’s training phase consisted of two potential target colors, with the exact color being unknown at the start of each trial. Thus, as with our unreliably pre-cued conditions, these training phase targets were associated with a history of instrumental information.

In conclusion, here we showed that observers were more likely to make a saccade to a task-irrelevant, physically non-salient distractor if it was rendered in a color that was associated with a history of providing instrumental information, relative to a distractor rendered in a color that was associated with a history of providing redundant information. Given that both kinds of distractors had equivalent histories as sought targets, this attentional difference must be due to their different information histories. The behavioral results in this study establish the plausibility of the information history hypothesis and provide a validated method for future work exploring the neural signature of information-driven attentional capture.

## Supplementary information

Below is the link to the electronic supplementary material.

Supplementary file1 (PDF 354 KB)

## Data Availability

Upon publication, data and analysis scripts will be available for download on the corresponding author’s website (www.attentionPerceptionDecision.com/DVCMG).

## Footnotes

1 A similar pattern of results was obtained when we restricted our analysis to the “first saccade” in each trial (see Online Supplemental Material (OSM)*Results* for details).

2 However, see OSM*Results* for a Bayesian analysis that models the full RT distributions and quantifies the impact of attending to each distractor type. In short, attending to distractors associated with instrumental information slowed test phase RTs more severely than did attending to distractors associated with redundant information.

3 For readers who prefer a frequentist analysis done at the trial level, these Bayesian results are corroborated by a generalized linear mixed-effects model fit with maximum likelihood estimation (fixed-effect slope: estimate = 0.0889, s.e. = 0.03, z = 2.836, p = 0.0046).

4 Reliably and unreliably pre-cued targets appeared an equal number of times in the training phase. That said, accuracy was significantly higher for reliably versus unreliably pre-cued targets, which means that observers were more likely to find and correctly report the orientation of the line inside the target when it was reliably pre-cued. Thus, the two conditions may not be perfectly matched on “history as a sought target” for all participants. This could be a problem if our hypothesis relied on the redundant-information-associated distractors exerting a stronger effect in the test phase than the instrumental-information-associated distractors. However, we predicted and found the opposite pattern.
