# Supplementary material for: Information-driven attentional capture
Source: Atten Percept Psychophys. 2025 Feb 19;87(3):721–7. doi: 10.3758/s13414-024-03008-z (PMC11965227; doi:10.3758/s13414-024-03008-z)
Supplement: Supplementary file 1 — Supplementary file1 (PDF 354 KB) [file 13414_2024_3008_MOESM1_ESM.pdf]

Supplementary Information

Supplementary Methods

Overview. Observers completed a modification of the protocol developed in Massa, Crotty, Levy & Grubb (2024), which is described in detail below. The only change was the elimination of trial-to-trial reward from the training phase, which was replaced with trial-to-trial feedback about the accuracy of the orientation discrimination judgement. The experiment consisted of 960 total trials, delivered in an approximately 1.5 hour session.

Observers. 131 observers participated in the study. Two observers participated in Massa *et al.,* (2024) and were inadvertently allowed to participate in the current study; both were excluded to ensure that no lingering associations impacted performance. Data from nine additional participants were excluded from all reported analyses for performing at chance accuracy levels in the training phase, test phase, or both. Chance accuracy was determined through simulated guessing for 480 trials (the number of trials in each phase of the study): We randomly drew (with replacement) from the set [0,1] 480 times, calculated the mean, repeated this process 10,000 times, and extracted the 95% confidence interval around the mean of the resulting distribution; 0.5438 marked the upper bound, and we used this as our inclusion cutoff[1]. These exclusions resulted in 120 participants for data analysis (age: mean = 19.47, range = 18 - 26; gender: 83F, 35M, 2 non-binary). This study received ethical approval by the Trinity College Institutional Review Board, and informed consent was obtained for each participant.

[1] With infinite data, this simulated guessing procedure will converge on 0.5.

Payment. All observers received $30 for participation. The monetary compensation scheme was the same for all participants, but individuals enrolled in an introductory psychology course were also able to count their participation toward a research participation requirement.

Training Phase. The task in the training phase was to search for a color-defined target (red, lime, cyan, or yellow[2]) and to report the orientation of the line contained inside (2AFC judgment: vertical or horizontal). Following a randomly selected period of fixation (400, 500, or 600 ms) and a 500-ms pre-cue, a visual search array of six color-defined circles (radii, 1.15 degrees of visual angle (DVA), line thickness = 6 pixels; 1 target, 5 distractors located at an eccentricity of 5 DVA) was presented on a gray background. The target was rendered in red, lime, cyan, or yellow, and the distractors' colors were chosen randomly, without replacement, from black, white, magenta, indigo, orange, and tan. Line segments inside the distractor circles were randomly and independently rotated 45° clockwise or counterclockwise of vertical (line thickness = 6 pixels). The search array remained onscreen until a response was made or until 800 ms, at which point the trial timed out. There was then an inter-stimulus interval of 1000 ms followed by 1500 ms of feedback (see *Training Phase Feedback* below for more details). Following each trial was an inter-trial interval of 1000 ms. There were 480 trials in the training phase, delivered as 5 blocks of 96 trials. Before beginning the training phase, observers completed 24 practice trials.

Pre-cues. Four distinct target colors were used during training (red, lime, cyan, yellow) to accommodate a within-subjects design that experimentally manipulated pre-cue reliability.

---

[2] Color names follow the web/X11 standards, see https://www.w3schools.com/Colors/colors_names.asp

Observers were provided with a pre-cue that was *reliable* (i.e., indicated the exact color of the upcoming target) or *unreliable* (i.e., indicated one of two potential target colors). For each observer, two target colors (e.g., red and lime) were always preceded by reliable pre-cues, and the remaining two target colors (e.g., yellow and cyan,) were always preceded by unreliable pre-cues. For reliable pre-cues, both fixation squares were rendered in the color of the subsequent target. For unreliable pre-cues, one fixation square was rendered in one of the unreliable cue colors, and the other fixation square was rendered in the remaining unreliable cue color; the exact spatial location (i.e., left square or right square) was randomly determined on each trial. The contingencies between color and pre-cue condition were counterbalanced across observers (*i.e.*, for half the observers, cyan and yellow were reliable pre-cue colors; for the other half of observers, red and lime were reliable pre-cue colors). Color-opponent pairs were preserved (i.e., red with lime, cyan with yellow).

Training Phase Feedback. We provided trial-to-trial feedback on the accuracy of the orientation discrimination judgement. If the response was correct, the word "correct" was displayed. If the response was made before the deadline but was incorrect, the word "incorrect" was displayed; if no response was made before the deadline, the words "too slow" were displayed.

Test Phase. The test phase replicated the methodology used in Massa *et al.*, 2024 and other relevant literature (e.g., Anderson & Halpern, 2017; Grubb & Li, 2018). The task was to search for a shape-defined target (a diamond among circles or a circle among diamonds, each appearing an equal number of times) and report the orientation of the line contained inside the target (vertical or horizontal). Observers were informed that color was irrelevant. The search array

remained onscreen until a response was made or until 1200 ms, at which point the trial timed out. Each element of the array was a unique color.

In half of the trials, one (and only one) of the distractor elements was rendered in a color that defined the training phase target (*i.e.*, red, lime, cyan, or yellow, with each appearing an equal number of times), referred to below as "information-associated distractors". If the distractor was rendered in a color that defined a training phase target that had been reliably pre-cued, we refer to these as "*redundant*-information-associated distractors". If the distractor was rendered in a color that defined a training phase target that had been unreliably pre-cued, we refer to these as "*instrumental*-information-associated distractors".

In the other half of the trials, none of the distractor shapes were rendered in a color that defined the training phase target. The target (i.e., the shape singleton) was never rendered in a color that defined the training phase target; the colors of the target and additional distractors were chosen randomly, without replacement, from black, white, magenta, indigo, orange, and tan. The target and information-associated distractor (when present) were equally likely to appear at all six locations in the search array. 1000 ms of accuracy-based feedback: "correct", "incorrect", and "too slow", was displayed for accurate responses, inaccurate responses, and missed response deadlines respectively, directly following the response window. Following each trial was an inter-trial interval of 1000 ms. There were 480 trials in the test phase, delivered as 5 blocks of 96 trials. Before beginning the test phase, observers completed 12 practice trials, with no information-associated distractors present.

Apparatus. The experiment was programmed in PsychoPy (Peirce & MacAskill, 2018) and run on a 3.0GHz Dual-Core Intel Core i7 Mac Mini; stimuli was displayed on 27.0" LED-Lit Dell

Gaming Monitor (model: S2716DG). Participants were seated in a darkened experimental testing room and kept their chins in a chin rest 70 cm from the monitor. Responses were collected with a Logitech F310 gaming controller. Participants were instructed to hold the controller with their right hand, to rest their index finger on the top right button, and to rest their thumb on the yellow Y button. The top right button was used to report horizontal judgements, and the yellow Y button was used to report vertical judgements.

Eye Tracking. Eye movements were recorded using an EyeLink 1000 infrared-video eye tracker (Eyelink, SR Research, Ottawa, Ontario). A 9-point calibration routine was performed before each experimental phase. We used the eyelinkReader package to analyze the EDF files in R Studio (https://github.com/alexander-pastukhov/eyelinkReader). For the saccade-based analyses below, we used the built-in Eyelink algorithm for saccade detection.

Data Analysis, Training Phase and Test Phase. Mean RT was calculated for correct trials only, and in line with convention (e.g., Massa *et al.*, 2024; Anderson *et al.*, 2011; Anderson & Halpern, 2017), RT distributions were trimmed to remove responses occurring 3 standard deviations above/below the condition mean. Task accuracy (proportion correct; trials in which the deadline was missed were counted as incorrect trials) served as a secondary dependent variable.

Data Analysis, Oculomotor Capture. Early work on oculomotor capture in studies of value-driven attentional capture used the proportion of first saccades to the target hemifield as the dependent variable (Anderson & Yantis, 2012), while later work has prioritized saccades and dwell time on the distractors themselves (e.g., Anderson & Kim, 2019). Here, we replicated our approach from Massa *et al.*, (2024) and localized saccades to one of six pie-shaped “wedges”

that emanate from the middle of the screen at 60 degree angles; the wedges are arranged such that one of the six potential target locations sits at the center of each wedge. If no saccades were detected on a given trial, one cannot determine the location of the saccade. Such trials were coded as NA, which led to their exclusion in the analyses reported below.

Data Analysis, Oculomotor Capture, Bayesian Modeling. We restricted our analyses to trials that contained an information-associated distractor. For each trial, we determined whether any saccade landed in the wedge that contained the information-associated distractor (binary outcome: yes=1, no=0).

We used generalized linear mixed-effects modeling with hierarchal Bayesian estimation (McElreath, 2020) to analyze the trial-level data. We modeled the binary outcome of each trial (*saccadeToDistractor*$_t$, where 1=any saccade made to distractor location, 0=none of the saccades made to distractor location) as a single draw from a binomial distribution with probability *p* (Model 1, Line 1).

$$\begin{aligned} \text{SaccToDistractor}_t &\sim \text{Bernoulli}(p) \\ \text{logit}(p) &= \beta_{0i} + \beta_1 \times \text{isIIA}_t \\ \beta_{0i} &\sim \mathcal{N}(\mu, \sigma) \\ \mu &\sim \mathcal{N}(0,1) \\ \sigma &\sim \text{Exponential}(1) \\ \beta_1 &\sim \mathcal{N}(0,1) \end{aligned} \qquad \textbf{(Model 1)}$$

We define *p* using a simple linear model that contains a "dummy" predictor for the distractor type on that trial, *isIIA*$_t$ (1 = is associated with *instrumental* information, 0 = is associated with *redundant* information, Model 1, line 2). Since *p* is a probability, it must always fall between zero and one. To ensure that this is the case, we use a "logit link function". Two

equations can help make this clear: the *logit* function and the *logistic* function (also known as the inverse logit). The *logit* function (Equation 1) converts a probability (denoted as *p*) into a real number on an unbounded scale (denoted as *x*). The *logistic* function (Equation 2) converts a real number on an unbounded scale (denoted as *x*) into a probability (denoted as *p*). At first glance, the construction of the linear model can be difficult to conceptually parse: $\text{logit}(p) = \beta_{0i} + \beta_1 \times \text{isIIA}_t$. So, consider the alternative but equivalent statement: $\text{p} = \text{logistic}(\beta_{0i} + \beta_1 \times \text{isIIA}_t)$, where $\beta_{0i} + \beta_1 \times \text{isIIA}_t$ substitutes for *x* in Equation 2.

$$\text{Logit function: } \text{logit}(p) = log\left(\frac{p}{1-p}\right) = x \qquad \textbf{Equation 1}$$

$$\text{Logistic function: } \text{logistic}(x) = \frac{1}{1+e^{-x}} = p \qquad \textbf{Equation 2}$$

According to our linear model, when the trial contains a redundant-information-associated distractor, logit(*p*) = β_{0i}. When the trial contains an instrumental-information-associated distractor, logit(*p*) = $\beta_{0i} + \beta_1$. In this model, β_1 quantifies the degree to which the presence of an instrumental-information-associated distractor changes the probability that a saccade is made to its location (a fixed effect), relative to a given individual's probability of a saccade to a redundant-information-associated distractor (a random effect, β_{0i}). The fact that we use a logit link function and have both fixed and random effects is what makes this a *generalized* linear *mixed*-effects model.

To estimate the posterior distributions for each parameter in Model 1, we use a hierarchal Bayesian approach with Hamiltonian Monte Carlo (McElreath, 2020). The random effects (i.e., β_{0i}) are modeled as coming from a normal distribution with mean μ and standard deviation σ.

(Model 1, Line 3). For all parameters, we used uninformative prior distributions so as not to bias the outcome in either direction (Model 1, lines 4-6). We used the *ulam* function in the *rethinking* package in R (McElreath, 2020) and ran 4 chains, each with 1000 iterations. The largest R-hat convergence diagnostic value for any parameter was 1.0019, which indicates successful mixing of the chains[3]. We then drew 2000 random samples from each posterior distribution using the *extract.samples* function in the *rethinking* package to calculate the values reported in the main manuscript. We used the lme4 package for R to compute the maximum-likelihood-based GLME reported in the main text (https://cran.r-project.org/web/packages/lme4/index.html).

Data Analysis, RT and Oculomotor Capture, Bayesian Modeling. We used linear mixed-effects modeling with hierarchal Bayesian estimation to model the time taken to make a correct response. We modeled the RT on each trial as coming from a lognormal distribution with mean μ and standard deviation σ (Model 2, Line 1). We set the mean and standard deviation parameters of the lognormal distribution equal to linear mixed-effects models that contained random effects ($\beta_{\mu 0i}$ and $\beta_{\sigma 0i}$) for each participant and fixed effects for the group ($\beta_{\mu 1}$ and $\beta_{\sigma 1}$, $\beta_{\mu 2}$ and $\beta_{\sigma 2}$, $\beta_{\mu 3}$ and $\beta_{\sigma 3}$). $\beta_{\mu 3}$ and $\beta_{\sigma 3}$ quantify the interaction between attending to a distractor (isAttended) and that distractor being associated with instrumental information (isIIA, Model 2, Lines 2 and 3). For all parameters, we used uninformative prior distributions so as not to bias the outcome in either direction (Model 2, Lines 5-9 and 11-15). We used the *ulam* function in the *rethinking* package in R (McElreath, 2020) and ran 4 chains, each with 1000 iterations. The largest R-hat convergence diagnostic value for any parameter was 1.0007, which indicates successful mixing of the chains.

[3] The models themselves are available with the data, as is a spreadsheet containing detailed information about each posterior distribution (mean, standard deviation, 95% credible interval, number of effective samples, and R-hat).

We then drew 2000 random samples from each posterior distribution using the *extract.samples* function in the *rethinking* package to calculate the values reported in the main manuscript. We used the posterior samples and Equation 3 below to calculate the mean of the lognormal distribution for each participant and each condition. We then took the mean across participants to obtain the sample-level estimate of RT in each condition.

$$
\begin{aligned}
\text{RT}_t &\sim \text{lognormal}(\mu, \sigma) \\
\mu &= \beta_{\mu 0i} + \beta_{\mu 1} \times \text{isIIA}_t + \beta_{\mu 2} \times \text{isAttended}_t + \beta_{\mu 3} \times \text{isIIA}_t \times \text{isAttended}_t \\
\sigma &= \beta_{\sigma 0i} + \beta_{\sigma 1} \times \text{isIIA}_t + \beta_{\sigma 2} \times \text{isAttended}_t + \beta_{\sigma 3} \times \text{isIIA}_t \times \text{isAttended}_t \\
\beta_{\mu 0i} &\sim \mathcal{N}(\mu_M, \sigma_M) \\
\mu_M &\sim \mathcal{N}(0,1) \\
\sigma_M &\sim \text{Exponential}(1) \\
\beta_{\mu 1} &\sim \mathcal{N}(0,1) \\
\beta_{\mu 2} &\sim \mathcal{N}(0,1) \\
\beta_{\mu 3} &\sim \mathcal{N}(0,1) \\
\beta_{\sigma 0i} &\sim \mathcal{N}(\mu_S, \sigma_S) \\
\mu_S &\sim \mathcal{N}(0,1) \\
\sigma_S &\sim \text{Exponential}(1) \\
\beta_{\sigma 1} &\sim \mathcal{N}(0,1) \\
\beta_{\sigma 2} &\sim \mathcal{N}(0,1) \\
\beta_{\sigma 3} &\sim \mathcal{N}(0,1)
\end{aligned}
\qquad \text{Model 2}
$$

$$\text{lognormal mean}(\mu, \sigma) = e^{\mu + \frac{\sigma^2}{2}} \qquad \textbf{Equation 3}$$

This analysis includes only trials in which an instrumental- or redundant-information-associated distractor is present. By necessity, it is further constrained to includes trials in which a saccade was made (at all, regardless of whether it was to an instrumental- or redundant-information-associated distractor, to a non-target stimulus, or to the target); and since it is

focused on modeling RTs, it includes only "correct" trials, as per usual in RT analyses. The linear model in this analysis has a coefficient for the distractor type on that trial (instrumental info vs. redundant info), a coefficient for the attentional outcome on that trial (distractor attended vs. distractor not attended), and a coefficient for the interaction between distractor type and attentional outcome. What this analysis asks is: *Does the amount of RT modulation that occurs when a distractor is attended, relative to not-attended, differ by distractor type?*

Preregistration. We started data collection for this project while we were writing up Massa *et al*., (2024), and we created a preregistration on the Open Science Framework (https://osf.io/tqfh9/). During the Massa *et al*., (2024) peer review process, we realized the importance of analyzing the oculomotor data at the trial level and focusing on saccades to the distractor rather than to the target. As a result, the analyses in the preregistration do not match what we report here (or in Massa *et al*., 2024). During the Massa *et al*., (2024) peer review process, we also realized that eye movements provided the most sensitive metric, but the preregistration referenced above contained a data collection stopping rule focusing on RT analyses, which we did not follow. We report these details here simply for transparency and not as a way to suggest that the analyses in the current manuscript were preregistered. That said, we set a maximum participant number of 120 in the preregistration and collected data until we reached that number in the current project.

Supplementary Results

Saccadic variability. Participants regularly made saccades while searching for the target in the test phase. We calculated the proportion of trials in which a saccade was detected during the presentation of the search array and found that saccades occurred in 77.44% of test phase trials. That said, there was considerable variability across participants: the percentage of trials in which a saccade was detected ranged from 0 to 100, with a median value of 90.10%. As we noted in Massa *et al.* (2024), a failure to detect a saccade could be due to (i) a saccade genuinely not being made, or (ii) eye data not being available. Eye data might not be available on a given trial for at least three reasons: (i) an inability to track at all during the initial calibration (for idiosyncratic reasons like anatomical features of the eye, contacts, glasses, eye-make-up, etc.), (ii) movement after initial calibration which resulted in a loss of the pupil, or (iii) a temporary loss of the pupil.

Results, Proportion of "First Saccades" to Distractor. For each participant, we calculated the proportion of trials in which the *first* saccade was made to (i) the location of an instrumental-information-associated distractor, (ii) the location of a redundant-information-associated distractor, and (iii), for the distractor-absent trials, the location of a "non-target" stimulus (we divided this number by 5 to account for the fact that there were 5 potential non-target stimuli that could be attended). A one-way ANOVA on these values, with distractor condition as a within-subject repeated-measures factor, indicated a significant main effect ($F(2, 236) = 36.54$, $p < 0.0001$). Follow-up paired t-tests demonstrated that both instrumental- and redundant-information-associated distractors increased the proportions of first saccades to their locations, relative to the distractor-absent condition (both ts > 6.14, both ps < 0.0001). Comparing

instrumental-information-associated distractors to a redundant-information-associated distractors revealed a marginally significant effect in the expected direction ($t(118) = 1.84$, $p = 0.0683$, two-tailed). We note, however, that considering only "first saccades" necessarily leaves out any modulation of overt attention that occurred when multiple saccades were made.

Table S1 Mean (standard deviation) for test phase conditions

	Distractor-absent	Reliably pre-cued	Unreliably pre-cued
Proportion of first saccades to distractor	0.1205 (0.03)	0.1606 (0.08)	0.1739 (0.07)

Results, RT and Oculomotor Capture, Bayesian Modeling. This analysis asked whether the amount of RT modulation that occurred when a distractor was attended, relative to not-attended, differed by distractor type. Indeed, attending to distractors associated with instrumental information (II-associated) slowed test phase RTs more severely than did attending to distractors associated with redundant information (RI-associated). The mean of $\beta_{\mu 3}$'s posterior distribution was positive (0.0165), its 95% credible interval did not contain zero (0.0036–0.029), and 99.5% of its density exceeded zero. To visualize the effect, we used the model results to calculate mean RT for each condition (attended and II-associated, 796.9ms; unattended and II-associated, 722.8ms; attended and RI-associated, 783.8ms; unattended and RI-associated, 722.5ms) and plotted the posterior distribution of the attention X information interaction (Figure S1). In short, attending to a distractor associated with a history of providing instrumental information induced a greater behavioral cost than did attending to a distractor with a history of providing redundant information.

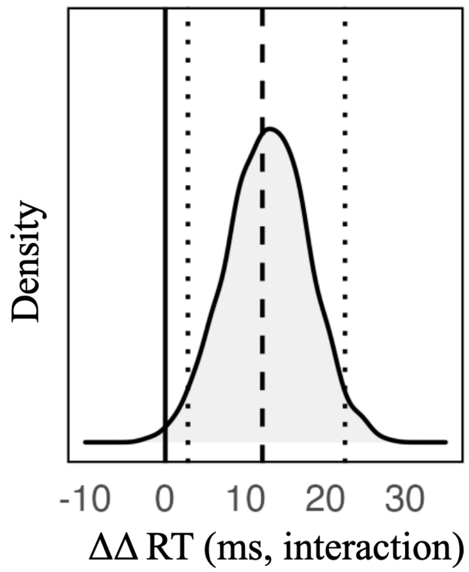

Figure S1. ***Behavioral cost of attending to a distractor associated with instrumental information.*** Dotted lines on posterior distribution indicate 95% credible interval, dashed line indicates the change in RT calculated directly from the data.
